# Matching Intent With Intensity: Implementation Research on the Intensity of Health and Nutrition Programs With Women's Self-Help Groups in India

**DOI:** 10.9745/GHSP-D-21-00383

**Published:** 2022-04-28

**Authors:** Avishek Hazra, Aikantika Das, Jaleel Ahmad, Shivani Singh, Indrajit Chaudhuri, Apollonius Purty, Audrey Prost, Sapna Desai

**Affiliations:** a Population Council, New Delhi, India.; b Project Concern International, New Delhi, India.; c Bihar Rural Livelihoods Promotion Society, Patna, India.; d UCL Institute of Global Health, London, United Kingdom.

## Abstract

**Introduction:**

In India, a large network of self-help groups (SHGs) implements interventions to improve women's and children's health and nutrition. There is growing evidence on the effectiveness of women's group interventions to improve health but limited information on implementation intensity, including how often groups meet, for how long, and with whom, despite this often being cited as a key factor for success. We aimed to assess the implementation intensity of large SHG-based health and nutrition interventions with rural, low-income women, to inform program design, delivery, and measurement.

**Methods:**

We synthesized process data from surveys, meeting observations, and process evaluations across 8 maternal and child health and nutrition interventions in India. We examined the implementation intensity of 3 common intervention delivery channels: group meetings, home visits, and community-level activities.

**Results:**

SHG members spent approximately 30 minutes in monthly meetings discussing health or nutrition. SHG dissolution or limited participation in meetings was a common challenge. Beyond group meetings, home visits reached approximately 1 in 3 households with an SHG member. Pregnant and breastfeeding women's participation in community events varied across interventions.

**Discussion:**

Interventions that aim to capitalize on existing networks of financial women's groups not specifically formed for health and nutrition objectives, such as SHGs, will need to have an implementation intensity that matches the ambition of their health objectives: substantial changes in behavioral or mortality outcomes are unlikely to be achieved with relatively light intensity. Interventions that require sustained interactions with members to achieve health outcomes need to ensure adequate community and individual outreach to supplement group meetings, as well as improved participation through more intensive community mobilization approaches. Evaluations of group-based interventions should report on implementation intensity to support the interpretation of evaluation evidence and to inform further scale-up.

## Abstract (precis)

Adding health interventions to women's groups primarily formed for financial purposes, such as self-help groups, is a widely used strategy to reach low-income women. An analysis of implementation intensity highlights the importance of ensuring that women's groups have sufficient time and population coverage to address health issues.

## Teaser

**Key Findings:**

- Women's self-help groups in 2 Indian states discussed health issues for approximately 30 minutes per month in group meetings, with wide variation in member participation across interventions.
- Home visits that aimed to reach women with health information outside of group meetings reached between 30%–40% of households with a group member. Women's participation in community events was low.
- Group-based interventions commonly employed a range of social and behavior change techniques that aimed to increase women's individual knowledge and build social networks.

**Key Implications:**

- Approaches to layering health activities onto existing women's groups should carefully consider the time available in group meetings to discuss health and calibrate intervention goals accordingly.
- Implementers and researchers should monitor, evaluate, and report implementation intensity of layered interventions, including group meetings, individual visits, and community-based events.
- Policy makers can explore a range of approaches to address health through working with groups, ranging from information dissemination to community mobilization. They should consider women's health priorities, time availability to participate in intervention activities, and the intensity required to improve health outcomes.

## BACKGROUND

Interventions with women's groups are an increasingly popular, potentially scalable approach to improve women's and children's health.^1^^,^^2^ Well-known group intervention models to improve health include: women's groups practicing participatory learning and action to improve maternal and newborn health; care groups for pregnant women and mothers; and sex worker collectives toward HIV prevention.^3^^–^^5^ Evidence of the effectiveness of some group intervention approaches have catalyzed large-scale investments, including on a national scale, in several low- and middle-income countries.^6^

In India, the government has invested in scaling up 2 approaches with women's groups to improve health. The National Health Mission supports government community health workers such as accredited social health activists (ASHAs) to engage women's groups in participatory learning and action to improve maternal and newborn health.^7^ Several evaluations in rural settings have reported reductions in neonatal mortality, including among the poorest families.^8^^–^^12^ Process evaluations identified adequate population coverage of groups (1 group per 500 population), inclusion of the most vulnerable, and relevance of issues being discussed to local communities as key components of program effectiveness.^13^ ASHAs currently implement this approach at scale in the states of Jharkhand and Madhya Pradesh, with planned expansion to 7 more states, and to issues beyond maternal and newborn health. The implementation processes associated with this approach have been studied in efficacy trials and at scale.^14^

In another approach, the National Rural Livelihood Mission supports the formation of women's self-help groups (SHGs), voluntary groups of 10–12 adult women who engage in joint savings, credit, and livelihoods activities. SHGs reached 50 million households by 2020, with the goal of reaching 70 million in the next 4 years.^15^ Although primarily a rural development intervention, SHGs are also viewed as a potential, wide-reaching “platform” to deliver additional services and information.^16^ For example, the Bill & Melinda Gates Foundation, UNICEF, and the World Bank have each supported different types of pilot interventions to improve women's and children's health and nutrition through SHGs.^17^^–^^19^ In 2017, the Ministry of Rural Development issued an advisory for SHGs to integrate information on food, nutrition, and health with water, sanitation, and hygiene into SHG meetings, an approach which is gradually being scaled across several states.^20^

There are 3 potential advantages of integrating health interventions into existing microfinance-based SHGs —commonly known as “layering”—in India.

1. Coverage: SHGs are widespread in many states, and their members are largely from low-income and vulnerable households that are also a focus of health interventions. For example, approximately half of rural households in Bihar and Jharkhand are covered by SHGs, and a population survey reported that nearly 30% of pregnant women/mothers with children aged 2 years and younger were SHG members in 3 additional states.^21^^,^^22^
2. Organizing structure: SHGs function according to established guidelines, which include weekly meetings and regular financial transactions, providing a ready forum to conduct additional discussions on health and nutrition. Groups are also federated at the village and cluster level, which supports collective activities and information dissemination beyond individual groups.
3. Due to their structure and functioning, SHGs may address underlying determinants of health by design, including financial security, decision making, and political participation.^22^^–^^24^

Taken together, these 3 features suggest that add-on interventions could result in a multiplier effect on health, nutrition, and well-being. Impact evaluations and observational studies indicate some improvements in health behaviors among SHG members through layered interventions.^1^^,^^2^^,^^25^^,^^26^ However, global evidence syntheses on women's groups have identified significant gaps in our understanding of **how** these interventions work—who participates, for how long, what do they do, and how often.^27^

Global evidence syntheses on women's groups have identified significant gaps in our understanding of how these interventions work.

Understanding the implementation intensity of these interventions is critical to identifying implementation features specific to women's groups that influence effectiveness, along with transferability and scalability in different settings.^28^^,^^29^ Hargreaves et al. define implementation intensity, or implementation strength, as a^30^:

*quantitative measure of the amount of inputs into, or activity to support, program implementation.*

Measures of intensity (e.g., frequency of contact with participants) vary based on an intervention's theory of change and envisaged processes. Interventions with SHGs to improve health and nutrition have several advantages but also specific challenges. The intensity of additional interventions with SHGs largely depends on the strength of the preexisting groups established to meet financial objectives; this objective defines the demographic profile of members and how frequently they meet.

Despite the widescale interest in improving health and nutrition outcomes using women's groups, including beyond India, evidence syntheses consistently highlight that relatively little is known about the intensity achieved in practice through add-on or layered health interventions.^2^^,^^25^^,^^27^ Implementation evidence is critical to refine design while contributing to analyses of transferability to other settings. This article aims to address this gap in the evidence base by synthesizing implementation evidence from interventions with SHGs to improve maternal and child health and nutrition in India to inform future program design, delivery, and measurement.

## METHODS

### Definition of Implementation Intensity for SHG Interventions

SHG-based health and nutrition interventions center on the presence of a preexisting group—a consistent, captive audience—and are premised on women learning new information and skills related to health and nutrition. The population coverage and active functioning of groups influence the overall community-level intensity of the intervention, supplemented by activities that extend beyond group meetings to reach individuals and community members. These typically include home visits, community-level meetings and events, and less commonly, supply-side interventions.^19^ Social and behavior change (SBC) techniques employed by interventions with SHGs include individual-level activities such as information dissemination, as well as group and community-level efforts to address underlying determinants of health, such as building social networks for advocacy.^31^

**FIGURE 1:**
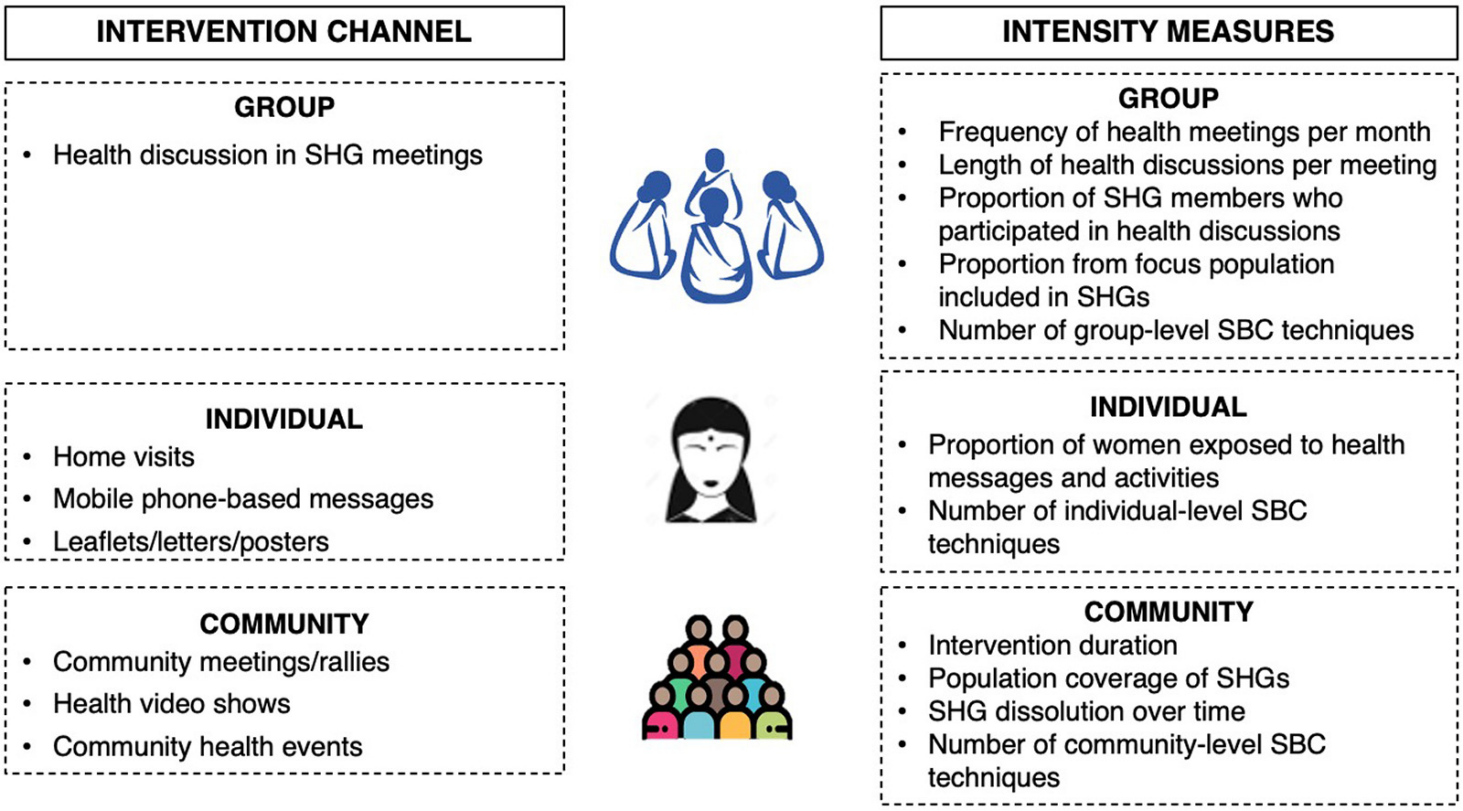
Intervention Channels and Corresponding Intensity Measures Within Self-Help Groups-Based Programs Abbreviations: SBC, social and behavior change; SHG, self-help group.

We drew from the literature on health and nutrition interventions with SHGs to identify 3 intervention channels relevant to implementation intensity: group meetings with SHG members; individual outreach; and community health activities. Figure 1 presents 3 main intervention components with our proposed indicators of implementation intensity. These included group discussions in SHG meetings (where only SHG members can participate), individual home visits by SHG members to meet women and family members, and other community-level events outside of SHG meetings where anyone (SHG members as well as nonmembers) can participate.

### Intervention Studies Included

We first identified studies from a 2020 published mixed-methods systematic review of studies on women's groups and health outcomes conducted in India.^1^ The review included quantitative and qualitative studies published between 2000 and 2019 that were peer-reviewed or gray literature and available in English. Of the 99 studies included, 44 were randomized or quasi-experimental trials that measured health outcomes on adult women or children aged younger than 5 years and 55 were observational or qualitative studies.

From the systematic review, we included 5 studies that met 2 inclusion criteria: (1) evaluated maternal and child health and nutrition interventions implemented with SHGs, and (2) reported indicators of implementation intensity, at minimum the meeting frequency and intervention coverage. The 5 studies that met these inclusion criteria reported on layered health interventions with 2 large SHG programs: (1) JEEViKA, a state-level government livelihoods program in Bihar under the National Rural Livelihood Mission, and (2) Uttar Pradesh Community Mobilization Program (UPCMP), led by a nongovernmental organization.^17^^,^^32^^–^^35^ In addition, we included 3 unpublished evaluations of health and nutrition interventions conducted under these 2 programs. For the analysis, we used data from the 5 published papers, and for the 3 unpublished papers, we report on the same indicators as in the published studies. In total, we included 8 experimental/quasi-experimental studies that included both household surveys and process evaluations.

The interventions had similar implementation approaches—health and nutrition discussions by trained facilitators in SHG meetings, home visits, and additional community-level events. The interventions included pilots in limited geographies to test the layering approach and subsequent implementation in larger geographies. Table 1 describes each intervention along with its geographical coverage, study participants, and the number of meetings observed. Three published studies did not report on meeting observation and/or have missing information about the length of health and nutrition discussion in the meetings. Two published and 2 unpublished studies did not report group dissolution information over the intervention period. The sampling procedure for household surveys was similar across the 8 included studies: representative samples (eligible women, as described in the last column of Table 1) were drawn from SHG households using a multistage approach to measure self-reported maternal and child health and nutrition practices. Each of the 8 studies included questions on respondents' participation in SHG meetings with health discussion, while 5 studies collected data on their exposure to health and nutrition messages outside of SHG meetings. Process evaluation data drew from household surveys, process monitoring data, and meeting observations.

**TABLE 1.** Description of Maternal and Child Health Interventions Implemented With Self-Help Groups in 2 States in India

Intervention	Coverage	Intervention Description, As Planned	# Meetings Observed	Survey Respondents
Parivartan^a^ pilot (2013–2014) to improve RMNCH behaviors^17^	Bihar, 8 Districts, 55 blocks	Groups formed to focus on health and nutrition along with savings and credit Structured health modules Weekly health discussion	Meeting observations not reported	Women SHG members with child aged 0–11 months
Parivartan pilot (2013–2016) to improve RMNCH behaviors^32^	Bihar, 11 districts, 64 blocks	Same interventions as above, with SHGs formed by the government to focus on savings, credit, and livelihoods, expanded into a larger geography with health and nutrition discussions held monthly	Meeting observations not reported	Women SHG members with child aged 0–11 months
JEEViKA multisectoral nutrition pilot (2016–2018) to improve anthropometry and dietary diversity^33^	Bihar, 1 district, 3 blocks	Maternal and child nutrition discussions in bi-monthly meetings Home visits, peer, and community meetings	30	Women from SHG households^b^ with child aged 6–23 months
JEEViKA-JTSP nutrition pilot^c^ (2017–2018) to improve nutrition behaviors (unpublished)	Bihar, 1 district, 4 blocks	Nutrition discussion in at least 1 of the 4 weekly meetings in a month Home visits and community events	60	Women from SHG households with child aged 6–23 months
JEEViKA Mobile Vaani pilot,^d^ JTSP (2017–2018) to improve RMNCH knowledge (unpublished)	Bihar, 1 district, 6 blocks	Interactive voice response based platform Information on nutrition, family planning, diarrhea, and entitlements in at least 1 monthly meeting Home visits and community events	172	Women from SHG households with child aged 0–23 months
UPCMP (2014) to improve RMNCH behaviors^34^	Uttar Pradesh, 1 district, 1 block,	Discussion on home-based newborn care and maternal health in 1 or 2 SHG meetings in a month	49	Women from SHG households
UPCMP (2015–2017) to improve RMNCH behaviours^35^	Uttar Pradesh, 37 districts, 120 blocks	Health discussion in at least 1 monthly meeting Home visits, community events	108	Women from SHG households with child aged 0–11 months
UPCMP (2015–2019) to improve RMNCH behaviors (unpublished)	Uttar Pradesh, 41 districts, 203 blocks	Same intervention as above, expanded to larger geography with more focus on household level discussion and community events (campaigns)	Meeting observations not reported	Women from SHG households with child aged 0–11 months

Abbreviations: JTSP, JEEViKA Technical Support Program; RMNCH, reproductive, maternal, newborn, and child health; SHG, self-help group; UPCMP, Uttar Pradesh Community Mobilization Program.

a Parivartan was a community mobilization project implemented by Project Concern International (PCI) to understand the efficacy of layering health and nutrition (HN) interventions onto the SHG platform to increase the adoption of HN behaviors among the most marginalized communities in 8 districts in Bihar.

b Either respondent or anyone from her family is an SHG member.

c JTSP is a technical assistance program to JEEViKA by PCI on HN integration in its livelihood framework in 101 blocks across 11 districts since 2015. JTSP identified 4 blocks of Nalanda district as learning blocks, in which all the HN interventions were pilot tested before scaling up to other geographies.

d JEEViKA Mobile Vaani pilot was implemented as a part of JTSP by Gram Vaani and PCI in 6 blocks of Nalanda district to assess the efficacy of a mobile-based voice-media communication platform in accelerating the pace and sustainability of behavior change and achieving higher outcomes in HN indicators.

The interventions we included in our analysis had similar implementation approaches—health and nutrition discussions by trained facilitators in SHG meetings, home visits, and additional community-level events.

### Analysis

We synthesized available data in 3 ways. First, we examined group and community-level intensity by compiling data on the frequency and length of SHG meetings on health, intervention duration, and group dissolution. Length of health discussion in meetings was based on direct observations, where reported. These meetings included scheduled health and nutrition meetings (typically the first meeting of every month) as well as regular SHG meetings. Next, we compiled data on women's exposure to health messages outside of SHG meetings. Finally, we drew on Kok et al.'s taxonomy to identify the number and types of social and behavior change techniques employed in interventions.^36^ The taxonomy categorizes 14 types of techniques that include individual-level approaches to improve knowledge, capacity, and skills and those aimed at addressing social and environmental conditions. We extracted these techniques from available intervention descriptions, process evaluations, and based on authors' experience with the specific interventions, we synthesized them into a heat map that categorizes individual and group/community-level techniques.

Table 2 describes indicators of implementation intensity for health and nutrition interventions at the group and community levels. Dedicated health meetings were typically held once or twice a month. The length of health discussions in meetings ranged from 10 to 27 minutes, as observed directly by researchers. Between 19% and 80% of group members reported attending a health meeting, suggesting that participation levels varied widely. The duration of health interventions with SHGs ranged from months to years. Where reported, between 24% and 33% of groups dissolved over the intervention period.

**TABLE 2.** Implementation Intensity of Health and Nutrition Interventions With Self-Help Groups at the Group and Community Level, 2 States in India

	Group Level			Community Level	
Study Details	Frequency of HN Meetings (Per Month), Based on Observations	Length of HN Discussion, as Observed Per Meeting, Minutes	Women Members (With Child Aged Younger Than 2 Years) Reported Participation in HN Meetings, %	Program Duration, Months	Group Dissolution Over Intervention Period, %
Parivartan pilot (2013–2014)^17^	4	Not reported	80.3^a^	12	27^b^
Parivartan pilot (2013–2016)^32^	1	Not reported	65.1^a^	36	3^b^
JEEViKA multisectoral pilot (2016–2018)^33^	2	10	Na	30	Not reported
JEEViKA-JTSP nutrition pilot (2017–2018)	1	27	26.9^c^	12	Not reported
JEEViKA Mobile Vaani pilot, JTSP (2017–2018)	1	20	18.7^c^	12	Not reported
UPCMP (2014)^34^	1	Not reported	37.9	4	Not reported
UPCMP (2015–2017)^35^	1	23	44.2^c^	24	24
UPCMP (2015–2019)	1	20	18.9^c^	48	33

Abbreviations: HN, health and nutrition; JTSP, JEEViKA Technical Support Program; UPCMP, Uttar Pradesh Community Mobilization Program.

a Based on calculation using questions on (1) participation in group meetings and (2) “Does your group ever discuss health topics related to pregnant women and young mothers?”

b Parivartan groups merged into JEEViKA during 2016–2017; many members joined JEEViKA SHGs.

c Based on calculation using questions (1) participation in group meetings and (2) “How many times in a typical month are health issues discussed during SHG meetings?”

**FIGURE 2:**
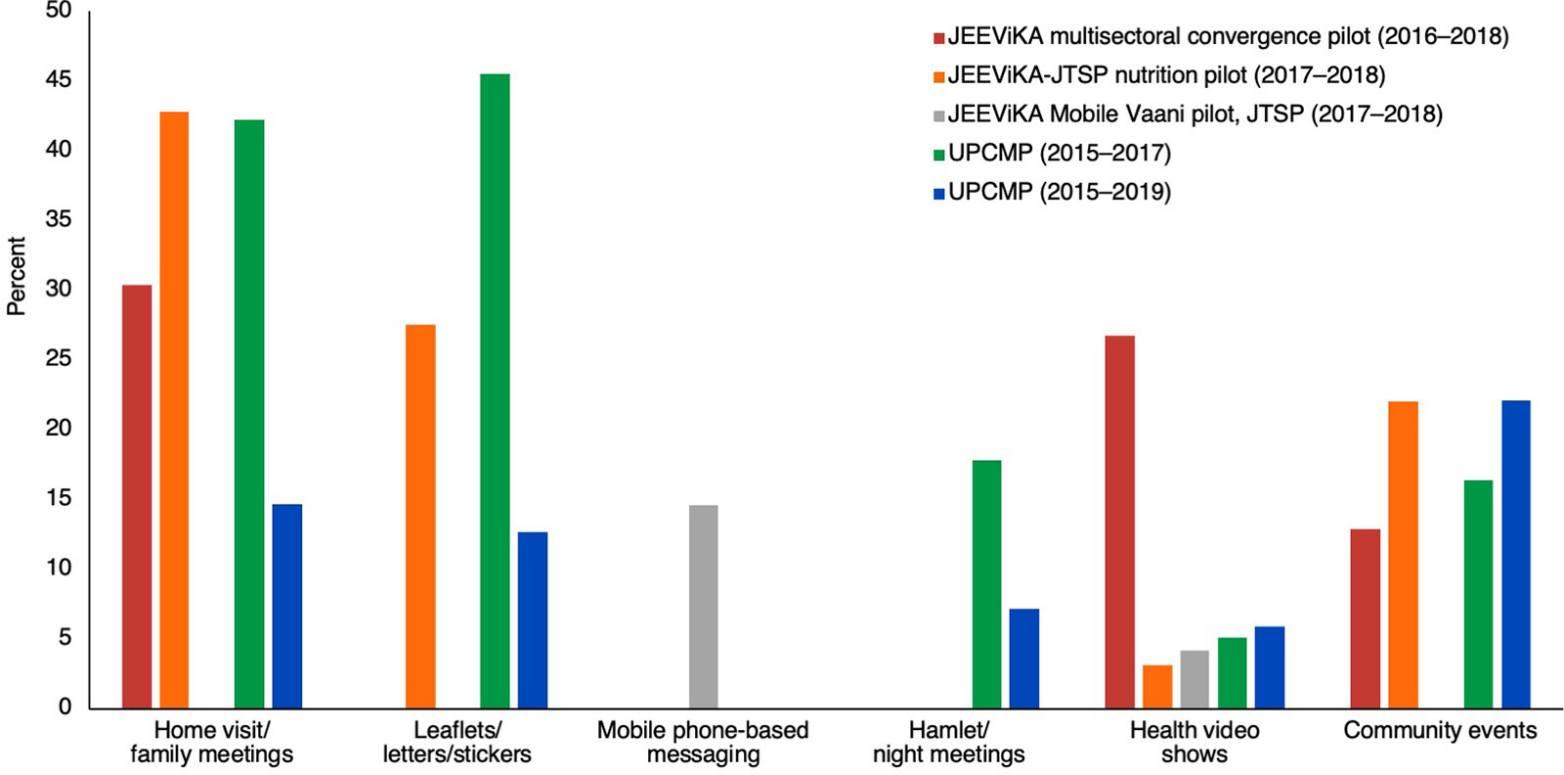
Percentage of Women Who Received Messages Outside of Group Meetings Among Women in Self-Help Group Households With Children Aged Younger Than 2 Years Abbreviations: JTSP, JEEViKA Technical Support Program; UPCMP, Uttar Pradesh Community Mobilization Program.

Figure 2 presents the percentage of women with children aged younger than 2 years in households having an SHG member who received health and nutrition information outside SHG meetings for the 5 studies that reported this information. In these interventions, intervention workers used home visits, family meetings, leaflets, letters, stickers with health messages, a mobile or interactive voice response system, community meetings, health video shows, and other community events to reach women and their families beyond SHG meetings. Home visits reached 30%–40% of women in SHG households and between 13% and 46% received additional information materials. However, less than 25% of women were reached through other channels such as community meetings.

**FIGURE 3:**
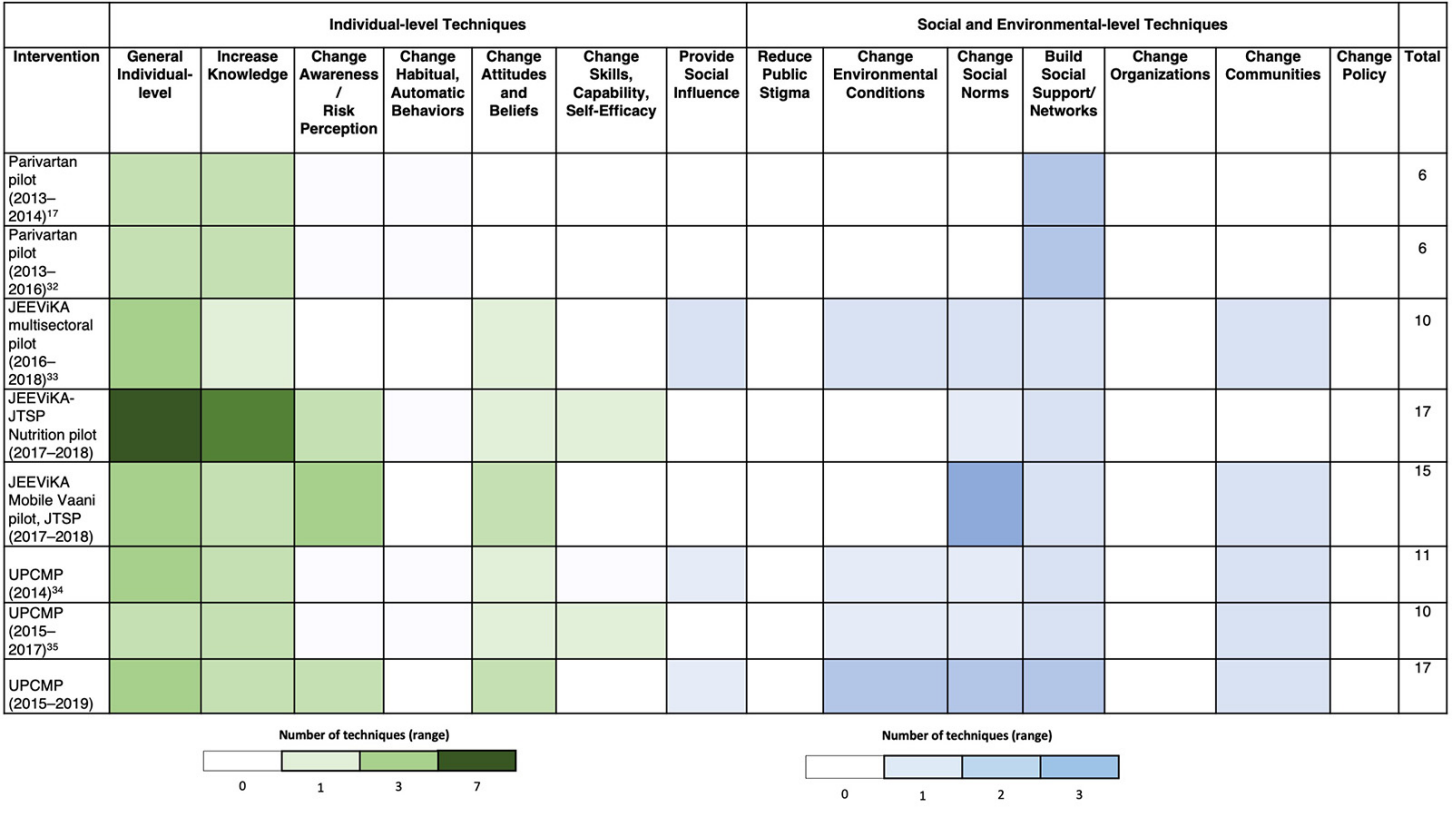
Social and Behavior Change Techniques Identified in Interventions Within Self-Help Group Programs in 2 States in India Abbreviations: JTSP, JEEViKA Technical Support Program; UPCMP, Uttar Pradesh Community Mobilization Program.

Figure 3 maps the social and behavior change techniques employed in SHG health and nutrition interventions. Two pilot interventions in Bihar focused on individual-level techniques. Other interventions, in both Uttar Pradesh and Bihar, used a broader range and higher number of individual and social and environmental-level techniques. The most common techniques used were to increase individual knowledge and build social networks.

## DISCUSSION

Our findings present a largely consistent picture of intensity achieved by health interventions with SHGs in Uttar Pradesh and Bihar, India. The programs studied had similar implementation models in which trained facilitators added health and nutrition interventions into large-scale SHG programs. Most interventions were implemented for at least 1 year, suggesting an intent to achieve health and nutrition outcomes through sustained contact with members, but available data from 2 studies indicated that between a quarter and a third of groups dissolved over the intervention period. In addition, SHG members spent approximately 30 minutes per month discussing health and nutrition. Interventions employed a similar number and range of social and behavior change techniques, consistent with the intent to reach women beyond the group meetings, along with family members and the wider community. Targeted activities, such as home visits, reached more than a third of households with an SHG member. As reported by women, community meetings and events were not well attended by the focus population of pregnant and breastfeeding women. Behavior change techniques varied according to the intervention's intended outcomes. For example, interventions that aimed to improve child dietary diversity placed greater emphasis on individual-level techniques, whereas maternal health interventions used more social and environmental techniques. No studies described the specific behaviors targeted by each technique.

Members' participation in health and nutrition meetings varied widely across interventions. Participation was higher in pilots of the layering approach^17^^,^^35^ compared to fully scaled-up programs, which may reflect less intensive program inputs when programs operate on a wider scale. Also, pilots had formed groups to address microfinance and health from the outset, which may have attracted women interested in health,^17^^,^^37^ whereas wider-scale implementation added health and nutrition into preexisting savings and credit groups. Consistently low levels of participation among mothers with children under 2 years in scaled-up health and nutrition meetings need further examination, to assess whether they are linked to irregular participation in SHG meetings in general or specific to their interest in the health and nutrition meetings.^38^ Notably, none of the studies reported population coverage of SHGs or proportion of relevant women within a group—key factors to understanding intensity at the population level. Government estimates suggest that between a third and half of low-income or socially vulnerable households have a member within a government SHG.^15^

Participation in meetings was higher in pilots of the layering approach compared to fully scaled-up programs, which may reflect less intensive program inputs when programs operate on a wider scale.

Our findings are consistent with a recent synthesis of enablers and barriers to implementing women's group interventions to improve health and nutrition.^1^ Most of these challenges are specific to SHG-based layering, which is premised on a preexisting, well-functioning, and wide-reaching network of SHGs to deliver information and impart skills. SHGs' primary focus and meeting purpose are financial transactions; as a result, studies report that facilitators had difficulties ensuring that sufficient time was available to discuss additional issues, including health and nutrition.^38^^,^^39^ Limited participation or discussions may also reflect a mismatch between member characteristics and choice of discussion topics. The mean age of government SHG members is 38 years,^40^ whereas some of the health and nutrition issues commonly addressed in these interventions were newborn care or breastfeeding. While women who are not pregnant or older members, such as mothers-in-law, may be household influencers, available data do not provide evidence of diffusion of messages from older SHG members to concerned women. Furthermore, dissolution of SHGs is a common challenge.^40^ Member drop-out or turnover, although not reported in studies in our sample, is common across group interventions in populations with high migration^41^—with some evidence from Uttar Pradesh that suggests poorer women are more likely to leave groups.^42^

### Implications

Our findings point to 3 priority areas to strengthen the delivery of health and nutrition interventions through microfinance-based women's groups.

First, programs will require a realistic assessment of time spent on health and nutrition in SHG meetings. The members spent 30 minutes per month discussing health, potentially because the group has limited time beyond primary objectives of savings and credit or health/nutrition topics were not of member's interest.^38^ Assessing members' needs and shaping discussions around their priorities may increase the time spent on discussing health. SHG members appear to have, at maximum, 1 meeting per month available beyond financial activities; health and nutrition goals should be defined or calibrated accordingly. An intervention that aims to improve awareness of health schemes, for example, may require considerably less intense contact—such as short, information sessions during a monthly meeting—while addressing child wasting or stunting requires in-depth interaction with families and services.

Second, the strength of the underlying group – regular meetings and sustainability over time– is likely a prerequisite before considering layering on additional interventions at scale. If groups are not meeting weekly as per their core principles, or groups are likely to dissolve, the platform itself may not be ready to absorb an additional load on women's time. While including nonfinancial topics may attract women to attend meetings, there is no evidence that groups with additional agendas have lower dissolution rates. Accordingly, add-on interventions should assess the readiness of the underlying group, as well as consider processes that support integration of new activities over time into the group's overall approach. The sustainability of SHGs poses another complexity to program implementation: older groups tend to conduct less regular group meetings, while newer groups meet more regularly but may require time to accept and adopt additional interventions.^40^

Strength of the underlying group is likely a prerequisite before considering layering on additional interventions at scale.

Third, where SHGs are not primarily composed of concerned women (i.e., pregnant women or new mothers), home visits or community events are the primary modes for reaching these women. Interventions may place a greater emphasis on community events and home visits, aligned with evidence on community interventions that have improved maternal health and nutrition in similar settings.^8^^,^^43^ Alternatively, SHG-based interventions may consider health outcomes beyond maternal and child health, aligned with group demographics and/or interest.

Further, the evidence base offers alternative approaches to engaging with groups to improve population health and nutrition. For example, an ongoing nutrition pilot in 3 states of India engages with SHG federations through participatory, community-based mapping of nutrition needs and active convergence across government departments to extend intervention reach. ^18^ In another approach, interventions to address malaria and dengue in urban and rural areas assigned SHG members a set number of houses in the community to monitor bed-net usage, actively working with groups as a conduit to reach the community in support of public health campaigns.^44^^,^^45^ Community mobilization interventions implemented at scale in India indicate the possibility of inviting other concerned women in the community to SHG meetings, which may improve participation in meetings as well. Critically, community mobilization interventions also offer the possibility of using problem-posing and problem-solving techniques in groups to ensure that participants have a say in deciding which health and nutrition problems are most prevalent in their context, and a role in deciding which intervention channels might best address these.^11^

Lastly, we have identified several areas for continued research on how women's groups improve health. Impact evaluations will benefit from describing and tracking implementation processes specific to women's groups, such as intensity and population coverage. While the studies in this synthesis reported on intensity to some extent, none captured population-level estimates of participation in the interventions. Research and analysis of why groups dissolve and the factors that influence member participation and retention will provide important insights. In addition, evaluations of behavior change interventions will benefit from analyses not just of the intensity of different techniques, but also whether the techniques were suited to the target behaviors.^32^ Accordingly, researchers may include an in-depth description of intended social and behavior change, using a typology such as Kok et al., along with an assessment of both intensity and suitability. While this synthesis focused on maternal and child health and nutrition outcomes, future research may consider how other health outcomes are influenced through group-based interventions.

### Limitations

Our analysis has some limitations. We did not link levels of, or variations in, program intensity to outcomes achieved since the required intensity of social and behavior change interventions depends on the intended outcome as well as specific behaviors. For example, a simple information dissemination campaign may be sufficient to improve awareness of schemes for institutional delivery, while reducing neonatal mortality requires extensive contact within and beyond the group to address individual beliefs and capabilities as well as social norms.^31^ Further, high rates of group dissolution resulted in a high risk of selection bias in several evaluations, limiting the ability to compare impact estimates. Our estimates of time spent on health meetings may be underestimated as it includes time discussing health in both scheduled and regular meetings. However, given the short time period observed in general, this limitation was unlikely to change our interpretation. Lastly, our findings are limited to 2, albeit large, states in India, given the lack of data from other settings.

## CONCLUSIONS

While SHGs or other women's group networks may provide a ready-made channel for health interventions, our findings indicate that interventions must reconsider how to improve the intensity with which concerned women can be reached. Low implementation intensity also suggests that observational evidence of improved maternal and child health behaviors among SHG households may depend on mechanisms outside of layering health and nutrition messages and activities onto group meetings, such as self-selection of members into groups.^26^ Our analysis illustrates the importance of collecting data on implementation intensity to re-calibrate interventions. At a minimum, future evaluations should ensure collection and reporting of process data, drawing from existing guidelines for group-based interventions,^46^ to report on implementation intensity aligned with the program's theory of change.^30^ In this case, low intensity of group meeting-based interventions, due to lack of time or low relevance of discussions to group members, may suggest the need for greater investment in community mobilization through participatory processes and home visits to reach concerned women. There is considerable evidence on how women's group interventions can improve health and nutrition outcomes, including sufficient population coverage, adequately intensive contact, behavior change techniques aligned with intended outcomes and community-wide engagement with and beyond groups.^1^ Accordingly, it may be time to reimagine how best to harness the potential of working with women's groups to improve health and nutrition—and invest in approaches that address population health needs with requisite intensity.

## Notes

**Peer Reviewed**

**First published online:** April 5, 2022.
